# Drivers of the In-Mouth Interaction between Lupin Protein Isolate and Selected Aroma Compounds: A Proton Transfer Reaction–Mass Spectrometry and Dynamic Time Intensity Analysis

**DOI:** 10.1021/acs.jafc.3c08819

**Published:** 2024-04-05

**Authors:** Cristina Barallat-Pérez, Michele Pedrotti, Teresa Oliviero, Sara Martins, Vincenzo Fogliano, Catrienus de Jong

**Affiliations:** † Department of Agrotechnology and Food Science, Wageningen University & Research, Wageningen, WG 6708, The Netherlands; ‡ Foundation Edmund Mach, San Michele all’Adige, TN 38098, Italy; § AFB International EU, Oss, LZ 5342, The Netherlands; ∥ Wageningen Food and Biobased Research, Wageningen University & Research, Wageningen, WG 6708, The Netherlands

**Keywords:** aroma compounds, release, binding, perception, lupin protein, proton transfer reaction of flight-mass spectrometry, time intensity, aqueous model systems

## Abstract

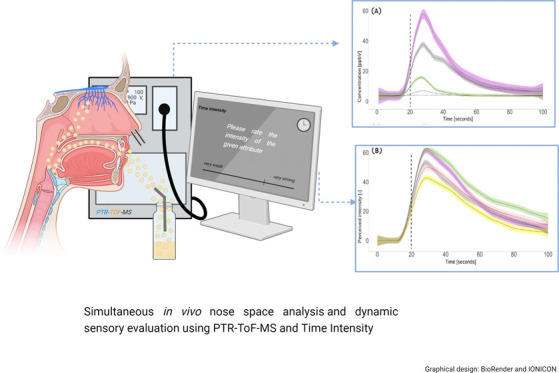

Plant proteins often carry off-notes, necessitating customized aroma addition. *In vitro* studies revealed protein–aroma binding, limiting release during consumption. This study employs *in vivo* nose space proton transfer reaction-time-of-flight–mass spectrometry and dynamic sensory evaluation (time intensity) to explore in-mouth interactions. In a lupin protein-based aqueous system, a sensory evaluation of a trained “green*”* attribute was conducted simultaneously with aroma release of hexanal, nonanal, and 2-nonanone during consumption. Results demonstrated that enlarging aldehyde chains and relocating the keto group reduced maximum perceived intensity (*I*_max__R) by 71.92 and 72.25%. Protein addition decreased *I*_max__R by 30.91, 36.84, and 72.41%, indicating protein–aroma interactions. Sensory findings revealed a perceived intensity that was lower upon protein addition. Aroma lingering correlated with aroma compounds’ volatility and hydrophobicity, with nonanal exhibiting the longest persistence. *In vitro* mucin addition increased aroma binding four to 12-fold. Combining PTR-ToF-MS and time intensity elucidated crucial food behavior, *i.e*., protein–aroma interactions, that are pivotal for food design.

## Introduction

Plant-based proteins have emerged as a popular substitute for animal proteins in creating innovative plant-based foods and beverages. While soybeans (*Glycine max*) and peas (*Pisum sativum* L.) have traditionally taken the spotlight,^1^ there is growing interest in exploring alternative protein sources. In Western Europe, lupin (*Lupinus angustifolius* L.) protein isolate (LPI) has recently gained attention because of its excellent interfacial properties. LPI forms weaker gels than soy protein isolate (SPI) upon heating, making it well-suited for high-protein beverage applications.^2^ Unlike soybean, lupin exhibits a milder bitterness due to its reduced saponin content.^3^ Despite being considered a potential protein replacement, lupin protein is characterized by cheese-like and sweaty profiles due to the presence of 2- and 3-methylbutanoic acid. Detectable but less pronounced cardboard-like, fatty, and green pepper-like off-notes (3-isopropyl-2-methoxypyrazine, (*E*)-non-2-enal and (*E, Z*)-nona-2,6-dienal) may also be present.^4^ These odor qualities can influence the sensory experience and affect its acceptability.

Various technologies are employed in the food industry to enhance (like cultivar selection and control of oxidation and temperature), remove (including soaking, thermal and enzymatic treatments), and mask (such as the addition of aroma) undesired aroma notes.^5^ Despite the array of available technologies, aroma addition offers an effective and customizable solution to improve the aroma of plant-based foods.

*In vitro* studies showed that aroma compounds can bind to proteins forming either weak and reversible bonds via hydrophobic, hydrogen, or electrostatic interactions or irreversible ones like covalent bonds.^6−9^ Protein–aroma binding may affect flavor perception by also regulating continuous release during consumption. Yet, the scenario differs under *in vivo* (dynamic) conditions during oral processing. During food consumption, aroma compounds must diffuse into the aqueous (saliva) phase and then transfer into the air phase of the oral cavity to enter the nasal cavity. Subsequently, the olfactory receptors perceive the aroma compounds and are ultimately sensed during oral processing.^10^ This recurring in-mouth event is known as retronasal olfaction.^11^ Due to the dynamic nature of oral processing and the rapidly changing conditions in the mouth, such as interactions between oral surfaces and foods, aroma compounds rarely reach an equilibrium state.^12^ Instead, oral processing involves a continuous state of equilibrium, reflecting a dynamic mass transportation phenomenon. The kinetic release of the aroma compounds from food systems is influenced by their molecular structure, thermodynamics, physicochemical characteristics, and the barrier to mass transfer from the food matrix to the air phase.^11−15^

Variables like the composition of the food matrix, conditions of consumption, and individual-specific parameters (i.e., chewing behavior and physiological characteristics)^15^ hold potential significance in modulating sensory perception. In-mouth interactions between salivary proteins and aroma compounds can alter flavored food perception.^10,15^ For instance, mucin proteins in saliva alter the distribution equilibria of aroma compounds, slowing their transport to the nasal cavity.^10,16,17^

For decades, flavor research has utilized dynamic techniques such as atmospheric pressure chemical ionization-mass spectrometry (APCI-MS) and proton transfer reaction-mass spectrometry (PTR-MS) to monitor volatile release. PTR-MS, coupled with a time-of-flight mass spectrometer (PTR-ToF-MS), is particularly suited for measuring *in vivo* aroma release from food products^18^ and, when complemented by dynamic sensory analysis like time intensity (TI) and temporal dominance of sensations, offers real-time insight into aroma release and perception.^19,20^ This combination has been employed to investigate the correlation between *in vivo* aroma release and perception in various products, including chewing gum,^18^ ice cream,^21^ mayonnaise,^22^ and chocolate hazelnut spreads.^23^ Despite extensive research using Gas Chromatography–Mass Spectrometry (GC-MS) and PTR-MS in the past decade on aroma compound release and their physicochemical properties,^24−29^ knowledge remains limited about plant protein-based systems, particularly with commercial food protein isolates.

For this purpose, this study delves into the drivers of the in-mouth interaction between lupin protein isolate and selected aroma compounds (hexanal, nonanal, and 2-nonanone) by coupling dynamic nose space PTR-ToF-MS and TI profiling. Lupin protein was selected for its promising potential in high-protein food product development and neutral taste and odor profile. Complementary *in vitro* analysis were performed with pig gastric mucin to investigate the interplay between mucin, protein, and aroma.

## Materials and Methods

### Materials

#### Lupin Protein Isolate

Lupin Protein Isolate 10600 was obtained from ProLupin GmbH (Grimmen, Germany). The manufacturer’s specifications indicated that the LPI contained 3% lipid and 91% protein. The protein batches were stored in a cool (10–15 °C), dry area away from light and air to minimize variability in the results. According to the manufacturer’s details, LPI was obtained through aqueous extraction and spray drying from seeds of the sweet blue lupine (*Lupinus angustifolius* L.) and had a taste ranging from neutral (pH 7.0) to grassy, accompanied by a grainy and flour-like odor.

The preparation of LPI stock solutions was done according to Barallat-Pérez et al.^7^ and adapted from Wang and Arntfield.^30^ Samples were prepared at an initial concentration of 2 wv% in Mili-Q water (pH 7.0). Subsequently, samples were vortexed for 10–20 s (3200 rpm, Genie II, Genie, Sigma-Aldrich, Florida, USA) and kept in a water bath (SW22, Julabo GmbH, Seelbach, Germany) for 20 min at 30 °C to provide a proper mixture of the protein solutions. Finally, the solutions were vortexed again (3200 rpm) for another 10–20 s to ensure homogeneity.

#### Aroma Compounds

Aroma compounds were selected based on their chemical class (aldehydes and ketones), structure (chain length and carbonyl group position), physicochemical properties (volatility, hydrophobicity, water solubility), and common use in beverages. Hexanal, nonanal, and 2-nonanone (Sigma-Aldrich, Zwijndrecht, The Netherlands) with a purity of ≥95% were chosen, meeting food-grade standards below their toxicity levels.

Each aroma compound was dissolved in MiliQ water (pH 7.0) at 10 mg/L in 100 mL amber glasses, following a modified version of Wang and Arntfield’s protocol.^30^ The stock solutions were then placed in a bath at 30 °C for 1 h to ensure optimal mixing.

Table 1 details the molecular structure and physicochemical properties of the selected aroma compounds.

**Table 1:**
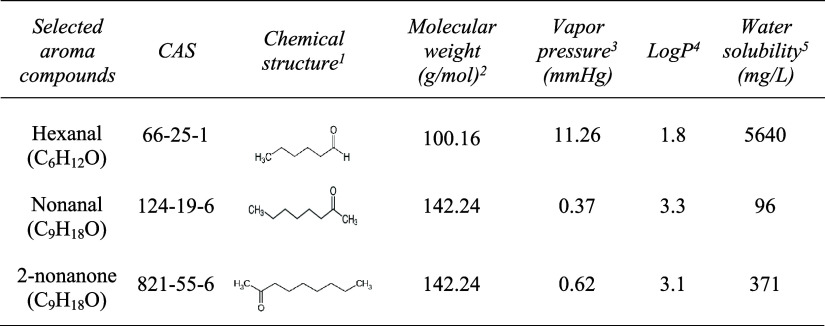
Physicochemical and Structural Features of the Selected Aroma Compounds^a^

a (1–5) Properties obtained from ref (31).

#### Creation of the Flavored Lupin Protein-Based Aqueous Model Systems

Seven aqueous model systems (three containing aroma but no protein, three containing both aroma and protein, and one containing protein but no added aroma) were prepared using MiliQ water (pH 7.0), protein (0 or 1 wv% LPI), and hexanal, nonanal, and 2-nonanone, following a modified protocol based on previous work by Barallat-Pérez et al.^7^ and Saint-Eve et al.^32^ The samples were incubated in a water bath, shaking at 125 rpm for 3 h before nose space analysis. Three hours proved adequate timing for achieving equilibrium.^30^Supporting Information Table S1 provides an overview of all samples.

A risk assessment was conducted to ensure safety, involving the identification of the main hazards and evaluation of the likelihood and severity of harm. The risk assessment demonstrated that there were no exposure risks involved in participating in the study. *In vitro* and *in vivo* pilot trials were performed to determine the optimal sample size (mL) and concentration (mg/L). Food applications typically involve concentrations in parts per billion (ppb) or parts per trillion (ppt).^33^ Thus, a final aroma concentration of 5 mg/L was selected, being consistent with comparable sensory studies^10,32,34,35^ This concentration is below the recommended maximum usage level according to FEMA GRAS 25th edition.^36^ A 10 mL aqueous model system, meeting food-grade standards, was spiked with aroma compounds, each added separately.

This study was exempted from the obligation to obtain ethical approval from the medical ethics committee overseeing human studies at Wageningen University. The study adhered to the principles outlined in the Declaration of Helsinki.

#### Other materials

Na_2_HPO_4_ and NaH_2_PO_4_·2H_2_O were analytical grade and purchased from Sigma-Aldrich, (St. Louis, Missouri, USA). Artificial saliva was made at 0.01 wv%, following the adapted version of van Ruth et al.^37^ Per 1000 mL the following ingredients were added: NaHCO_3_ (5.208 g), K_2_HPO_4_·3H_2_O (1.369 g), NaCl (0.877 g), KCl (0.477 g), CaCl_2_·2H_2_O (0.441 g), pig gastric mucin (M) (2.160 g), and NaN_3_ (0.5 g), provided by Sigma-Aldrich.

### Methods

#### Focus Group Discussions

Focus group discussions were conducted before the sensory evaluation to gauge consumer preferences for the three chosen protein isolates in aqueous solution: SPI, LPI, and pea protein isolate (PPI). The recruitment targeted regular consumers (*n*= 40) of plant-based beverages from Wageningen University. Consumers were asked to select the preferred protein based on the overall taste and odor. LPI emerged as the preferred candidate for the study, with 52.5% of the panelists choosing it over PPI or SPI (Figure S1).

#### Subjects

Ten European female subjects (26 ± 2 years, mean ± SD) were recruited from Wageningen University for this study. The selected criteria included nonsmoking status, absence of swallowing disorders, no allergy to lupin, and no use of dental braces. Saliva flow rate (0.145 ± 0.1 g/min, mean ± SD) and mouth volume (75 ± 8.5 g water, mean ± SD) were measured to complement the understanding of *in vivo* aroma release.^15^ All participants provided informed, with written consent under the European Data Protection Regulation (UE 679/2016), and received financial compensation for their participation.

#### Sensory Training

Participants underwent three training sessions to ensure optimal performance during the study. Additional data are available in the Supporting Information, Figure S2, which offers details regarding the initial attributes description during the first training session. Samples were generally described as fruity, synthetic, herbal, lemongrass, sweet, cucumber, grass, green, bitter, and grain-like (Figure S2). After reviewing panelist descriptions (Figure S2) and the odor/taste description found in the literature,^38,39^ a consensus for all samples was achieved, resulting in the selection of the attribute “green”. “Green” was defined as “reminiscent of grass and vegetables, with a slight pungency, accompanied by hints of fruitiness and freshness”.^38^ In the first session, they familiarized themselves with the samples and learned the definition of the selected “green” attribute.

In a second training session, participants learned about the use of EyeQuestion software (version 5, Logic8 BV, Est, The Netherlands) and the sensory methodology.

In the last session, the panelists became acquainted with the nose space pieces on their insertion into the nostrils and the consumption protocol (i.e., swallowing while breathing through the nose space pieces) to instill a sense of fearlessness and comfort in them.

#### Simultaneous *In Vivo* Nose Space Analysis and Dynamic Sensory Evaluation

The protein–aroma binding was assessed using PTR-ToF-MS and TI concurrently. Subjects followed a standardized drinking protocol to reduce the variability. *In vivo* nose space experiments were conducted with a high-sensitivity PTR-QiToF-MS (Ionicon Analytik, Innsbruck, Austria)^40^ with a drift tube temperature of 100 °C, voltage of 900 V, and pressure of 460 Pa, resulting in a field density ratio (E/N) of 133 Td. The volatile compounds present in the nose space were introduced into the system through a PEEK capillary line (1/16″ OD, 0.01″ ID, 0.32) heated to 100 °C with a flow rate of 40 mL/min. The mass resolution (*m*/Δ*m*) was at least 4800, and data were collected for the mass range *m*/*z* 20–25.^40^

**Figure 1:**
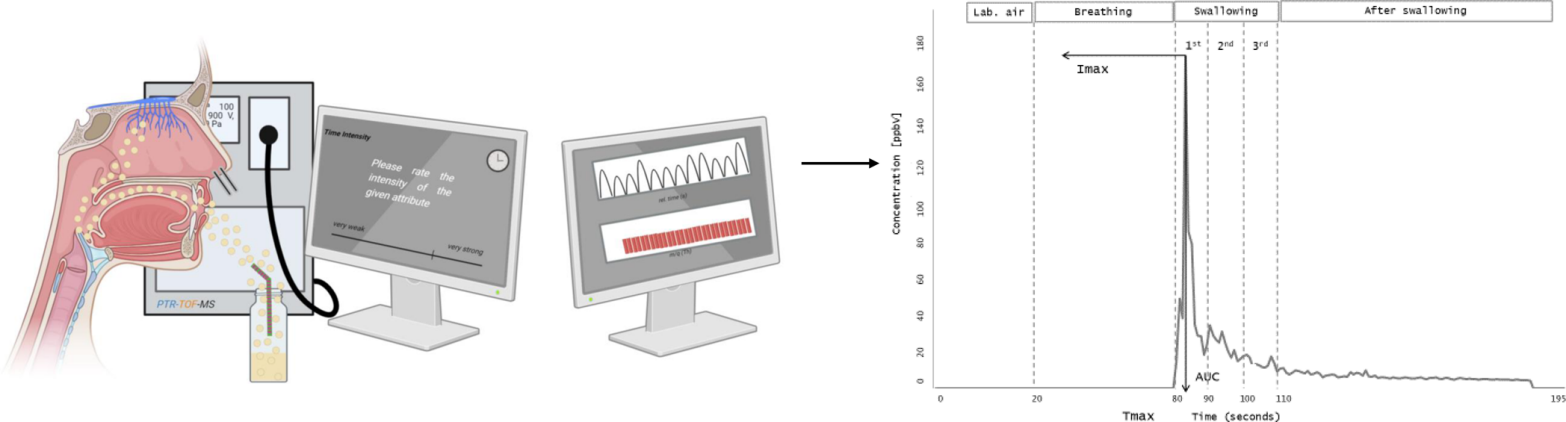
Graphical overview of the simultaneous assessment of aroma release and perception.^41,42^

Figure 1 illustrates the simultaneous assessment of aroma release and perception by PTR-ToF-MS and TI. As seen in Figure 1, first, the background signal was measured for 20 s. Each participant inserted two Teflon nose space pieces (6.8 mm diameter and 6.4 cm length) into their nostrils, connected to a heated (100 °C) N.A.SE device (Ionicon Analytik, Innsbruck, Austria). They then breathed regularly for 1 min to establish a breath baseline.

Dynamic sensory evaluation was performed using TI^43^ (EyeQuestion software). Subjects were prohibited from consuming food or beverages (except water) for 1 h before the test. Samples were coded with three-digit random numbers and served at 25 ± 5 °C in a 20 mL clear GC-MS glass vial (75.5 mm × 17.5 mm) closed with a screw metallic cap. The samples were randomly assigned to participants and over the evaluation sessions to ensure unbiased testing conditions. This means that each participant and session received a random selection of samples with no predictable order. Samples were offered one by one for consumption and evaluated in triplicate. The panelists rinsed their mouths between samples with water and unsalted crackers. Although rinsing may remove some residual material, this method carries the slight risk of inducing carry-over.

Before the start of the TI sensory evaluation, the operator unscrew the glass vial and introduced the straw. Subsequently, the panelists were ready to commence the measurements. The subjects sipped through a straw, held the sample in their mouth for 10 s, and then swallowed. After 10 s, the subjects swallowed again. In some cases, a third swallow was needed. Subjects rated the attribute intensity using a 100 mm unstructured line scale with anchors from “very weak” to “very strong” (Figure 1). To avoid the halo-dumping effect and sensory fatigue, and to maintain the subjects’ interest, a maximum of six samples were tested per session.^44^

#### Preparation of the Gas Chromatography–Mass Spectrometry Samples

A modified method based on Barallat-Pérez et al.^7^ and adapted from Wang and Arntfield^30^ was employed for Static Headspace GC-MS (HS-GC-MS). Samples consisted of three different combinations: LPI + aroma, mucin + aroma, and LPI + aroma + mucin. The concentrations used were 1 wv% LPI, 5 mg/L aroma, and 0.01 wv% mucin. Reference samples included buffered LPI and mucin solutions without an added aroma. Vials were sealed and incubated in a water bath shaker (SW22, Julabo GmbH, Seelbach, Germany) at 30 °C and 125 rpm for 3 h before headspace analysis. Samples were prepared in triplicate.

#### Data Collection, Analysis, and Processing

##### Time Intensity Data Treatment

The TI data obtained were defined by the parameters: area under the curve (AUC), which represents the total perceived intensity over the entire consumption time; maximum perceived intensity (*I*_max_), defined as the highest peak of perceived intensity within a sample; and time to reach the maximum intensity (*T*_max_), which corresponds to the time to reach *I*_max_. The data were then averaged per panelist (*n* = 10, in triplicate) and further analyzed. Smoothing of TI curves was done via the *geom_smooth* function in the *ggplot2* package of R software, version 4.2.1.

##### PTR-MS Data Treatment and Peak Selection

PTR-ToF-MS data was treated with the PTR Viewer software (version 3.4.2.1, Ionicon Analytik, Innsbruck, Austria) for internal mass axis calibration, mass peaks selection, and nose space concentrations extraction (parts per billion by volume; ppbV). In this study, *m*/*z* 101.103 was specifically chosen for hexanal while for nonanal, and 2-nonanone the *m*/*z* 143.158 was selected. The primary main fragments of hexanal (*m*/*z* 83.055) and nonanal/2-nonanone (*m*/*z* 125.142) were selected based on comprehensive reviews^45−47^ and prior piloting, i.e., HS analysis of the samples, which revealed the fragmentation pattern of each compound. Accordingly, absolute quantification was derived by summing the obtained values corresponding to the molecular ion fragments.

The results were presented as the mean for a sample size of *n* = 10, in triplicate. For each selected mass peak, averaged release curves (concentration in ppbV) were plotted against time (s) for each sample combination. Smoothing of PTR-ToF-MS curves was done via the *geom_smooth* function in the *ggplot2* package of R software, version 4.2.1.

##### Aroma Lingering and Decay

To investigate the interaction between aroma molecular structure and physicochemical properties on lingering and decay rates, calculations were performed. Aroma lingering refers to the persistence of aroma in the mouth after product consumption. Aroma lingering was calculated as the average of n = 10 individuals tested in triplicate. Each parameter was averaged for all subjects, all replicates, per second, and samples after the third and last swallow until the end of the test. The rate of change (decay rate) for both the PTR-ToF-MS and TI data was calculated for each sample combination. Data were fitted to an exponential curve, and calculated using eq 1:^48^
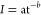
1where *I* was the intensity at time *t*. The two parameters obtained from the fitting represented the intensity at the beginning (*a*) and the decay rate (*b*) of the aroma compounds.^48^

#### Binding Measurement and Calculation

Protein–aroma–mucin binding and interaction were assessed by HS through GC-MS (Agilent- 7890A GC coupled to an Agilent 5975C with triple-axis detector MS, Agilent, Amstelveen, The Netherlands) following a modified method from Wang and Arntfield^30^ and adapted from Barallat-Pérez et al.^7^ Aroma binding to proteins, expressed as a percentage in the absence and presence of protein, was calculated (eq 2):^30^
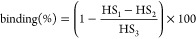
2where HS_1_ represents the abundance of the flavored protein-based aqueous solution in the headspace. HS_2_ and HS_3_ denote the abundances in the headspace without aroma (HS_2_) or protein (HS_3_).

Aroma binding to mucin was calculated and expressed in %, in the absence and presence of mucin (eq 3):
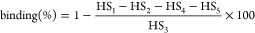
3

In this equation, HS_4_ (mucin solution + buffer) represents the headspace abundance without aroma, while HS_5_ (protein solution + mucin solution) indicates the headspace abundance of the protein-based mucin solution.

##### Statistical Analysis

For the statistical analysis, GraphPad (Prism 9.3.1471) and RStudio 4.2.1 (Boston, Massachusetts, USA) were utilized to conduct an Analysis of Variance (two-way ANOVA) for each sample combination and determine AUC, *I*_max_, and *T*_max_ parameters. Tukey posthoc tests were then performed to assess significant differences (*p* < 0.05) between each sample combination.

## Results and Discussion

### Effect of Aroma Molecular Structure on the *In Vivo* Aroma Release and Perception

#### Chain Length

**Figure 2:**
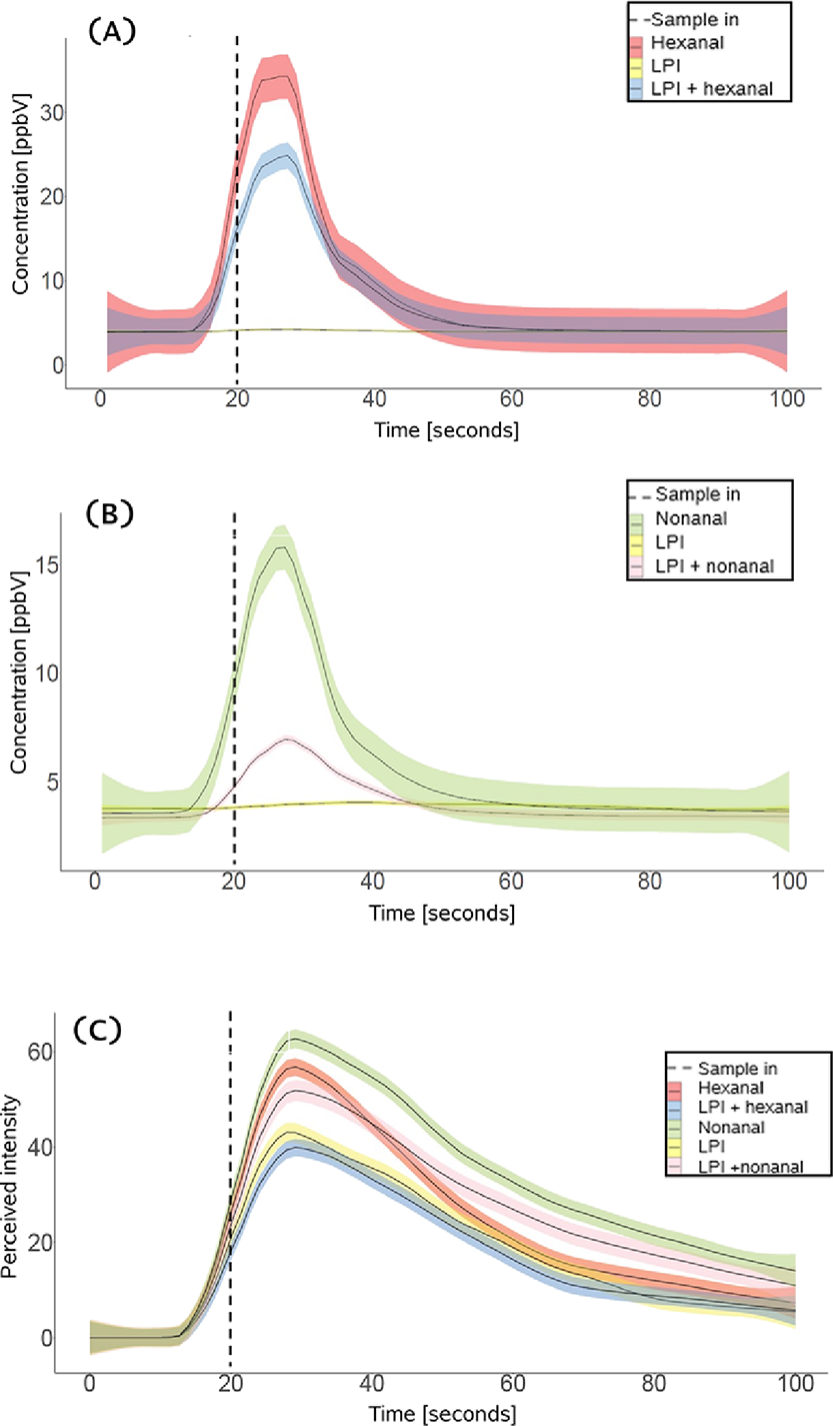
Averaged and standard error of (A) *in vivo* hexanal release (*m*/*z* = 83.093 + 101.103), (B) *in vivo* nonanal release (*m*/*z* = 143.158 + fragments), and (C) sensory perceived intensity (hexanal and nonanal) curves during drinking and after swallowing for aqueous model systems containing lupin protein isolate (LPI) only or LPI and hexanal or nonanal (*n* = 10 subjects, in triplicate). Scales are adjusted to their maximum responses for better data presentation.

The influence of carbonyl chain length (hexanal, (C6), and nonanal, (C9)) on the *in vivo* aroma release and dynamic sensory perception of the “green” attribute in the aqueous model systems is depicted in Figure 2 A-C. An overview of the *in vivo* aroma release parameters (AUC_R, *I*_max__R, and *T*_max__R) and the dynamic sensory “green” perceived intensity parameters (AUC_S, *I*_max__S, and *T*_max__S) can be found in Table 2.

**Table 2:** Summary of Parameters (Mean ± SE) Describing the *In Vivo* Hexanal, *In Vivo* Nonanal, *In Vivo* 2-Nonanone, and Dynamic “Green” Perceived Intensity for Flavored Lupin Protein-Based Aqueous Model Systems^a^

	LPI				LPI + 2-nonanone		LPI + nonanal		LPI + hexanal		2-nonanone		nonanal		hexanal	
	*m*/*z* = 101.103		*m*/*z* = 143.158		*m*/*z* = 143.158		*m*/*z* = 143.158		*m*/*z* = 101.103		*m*/*z* = 143.158		*m*/*z* = 143.158		*m*/*z* = 101.103	
AUC_R	251 ± 11	d	243 ± 10	d	1261 ± 116	a	271 ± 11	d	581 ± 41	bc	1515 ± 116	a	401 ± 36	cd	727 ± 69	b
*I*_max__R	6 ± 2	d	5 ± 1	d	132 ± 15	a	16 ± 2	d	142 ± 18	bc	209 ± 28	a	58 ± 13	cd	207 ± 32	b
*T*_max__R	12 ± 2	b	26 ± 3	a	8 ± 1	b	7 ± 1	b	5 ± 4	b	9 ± 1	b	10 ± 2	b	6 ± 1	b
AUC_S	1800 ± 268			ab	2155 ± 295	ab	2127 ± 287	ab	1555 ± 211	b	2245 ± 209	ab	2735 ± 248	a	2099 ± 222	ab
*I*_max__S	51 ± 5			b	62 ± 5	ab	60 ± 5	ab	54 ± 5	b	70 ± 4	ab	71 ± 4	a	67 ± 4	ab
*T*_max__S	6 ± 2			a	7 ± 2	a	10 ± 3	a	9 ± 2	a	6 ± 1	a	10 ± 3	a	7 ± 1	a

a Letters denote significant differences (*p* < 0.05). Treatments with the same letter are not significantly different.

The *in vivo* nose space release curves for lupin-free samples and those with nonanal and hexanal (Figure 2A,B) exhibited distinct release profiles, despite belonging to the same chemical class. As shown in Table 2, increasing the chain length led to a significant decrease in AUC_R and *I*_max__R by 44.89 and 71.92%, respectively. No significant differences were observed in the *T*_max__R values. The decrease in AUC_R indicates reduced nonanal release over time, while the decline in *I*_max__R may suggest a decrease in the maximum perceived intensity.

Aroma release in food systems is influenced by both thermodynamic (aroma compound volatility) and kinetic factors (mass transfer resistance from liquid to air phase),^13^ characterized by nonequilibrium conditions.^14^ Oral processing involves continuous equilibrium changes, reflecting a dynamic mass transport. Despite hexanal’s hydrophilic nature, it is thirty-fold higher volatility compared to nonanal (see Table 1), suggesting that it is the primary driver for aroma release.

Protein inclusion led to a 20.06% decrease in AUC_R for LPI + hexanal and a 32.37% decrease for LPI + nonanal (Table 2). Similarly, *I*_max__R decreased by 30.91% for LPI + hexanal and 72.41% for LPI + nonanal, indicating weaker aroma detection compared to samples without protein. Protein–aroma interactions may alter aroma release kinetics,^6^ resulting in slower release and potentially reducing maximum perceived intensity. The protein’s surface contains “hydrophobic binding sites” where small ligands, like aroma compounds, may bind. Aldehydes can bind to proteins through reversible or irreversible mechanisms, such as cysteine-aldehyde condensation reactions and Schiff base formation under certain conditions (e.g., pH 6–10), forming strong amide linkages.^9^

Despite clear binding effects observed in the *in vivo* aldehyde release results (Figure 2 A,B), dynamic sensory evaluation (Figure 2 C) showed discrepancies. In protein-free samples, increasing chain length slightly increased both AUC_S and *I*_max__S by 30.31 and 6.24%, respectively (Table 2). Upon protein addition, AUC_S decreased by 25.91 and 22.25%, while *I*_max__S decreased by 25.92 and 15.23%, respectively (Table 2).

Unsurprisingly, discrepancies between methodologies are common,^18,22,23,50^ with many analytical techniques lacking the sensitivity of the human nose.^18^ In Figure 2A,B, hexanal and nonanal were not detected in unflavored samples *in vivo*. These two aroma compounds are linked to green and grassy notes (Figure S2). Faint green notes were found to a certain extent in unflavored samples (Figure 2 C). Additional insights were gleaned from sensory evaluation (see Figure S3) to understand lupin off-notes. Light green, grain-like, cereal, butter, fruity, barley, grassy, sour, and lemon-like were the most commonly selected attributes to describe lupin (Figure S3). Even though lupin is mildly associated with green notes, its green citation proportion is significantly lower compared to the samples lacking protein (*e.g.,* hexanal, nonanal, and 2-nonanone) and the flavored-protein samples (Figure S3).

Despite the performance of three training sessions, the variation observed in release and perception (Figure 2A,B and Figure 2 C, respectively) may be linked to insufficient training sessions related to the definition of the trained “green” attribute. This could have resulted in a dumping effect or hasty responses that do not accurately consider the agreed definition for the selected attribute. However, it is imperative to acknowledge the potential for a carry-over effect. Remaining traces from a previous sample may have persistently appeared in subsequent measurements, affecting the score of the trained “green” attribute.

Establishing a direct link between *in vivo* aroma release and perception is challenging due to food matrix effects and interindividual differences, which often play a significant role.^18,50^

#### Reactivity and Position of the Carbonyl Group on the Alkyl Chain

**Figure 3:**
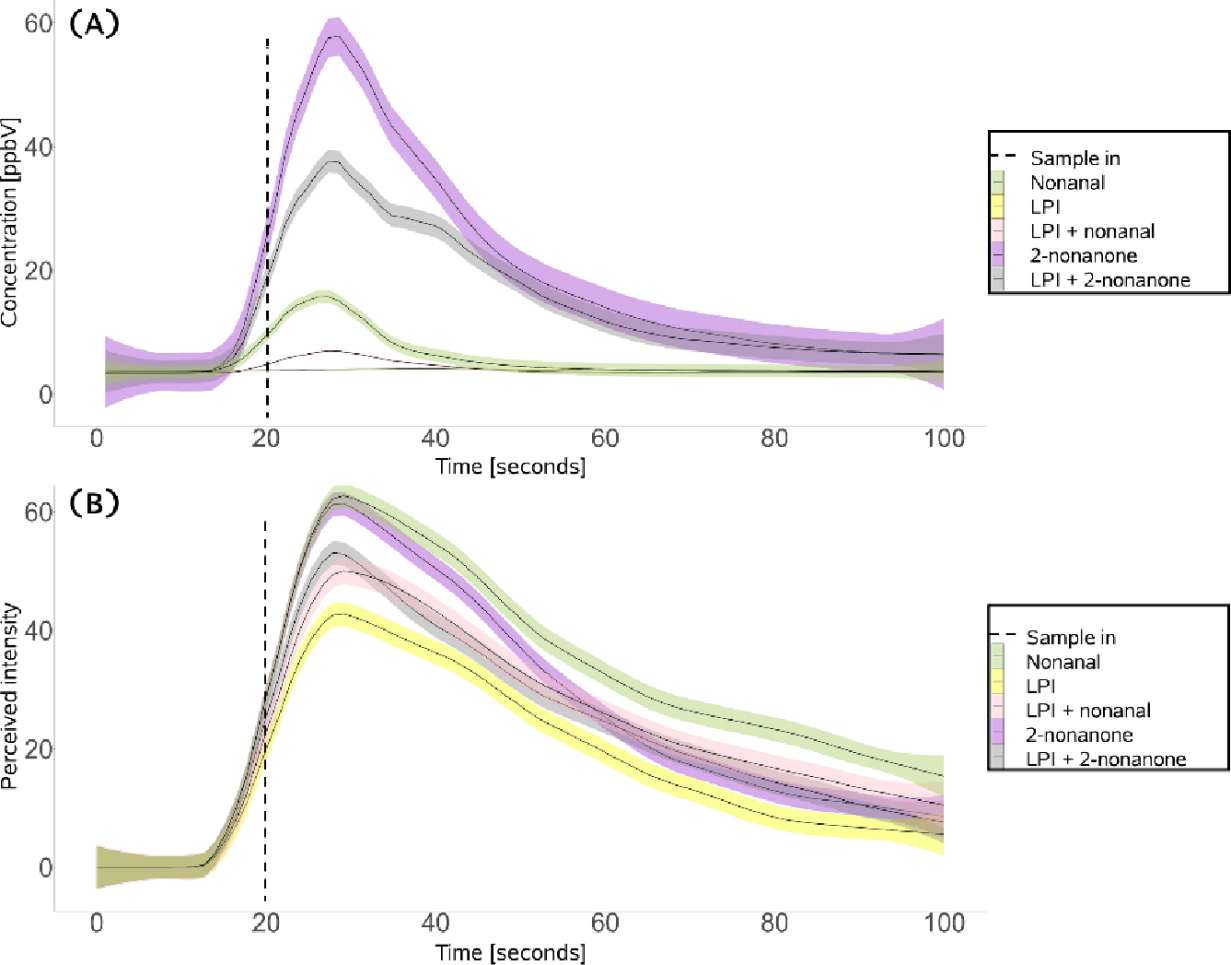
Averaged and standard error of (A) *in vivo* nonanal release and *in vivo* 2-nonanone release (*m*/*z* = 143.158 + fragments), and (C) sensory perceived intensity (nonanal and 2-nonanone) (B) curves during drinking and after swallowing for aqueous model systems containing lupin protein isolate (LPI) only or LPI and nonanal or 2-nonanone (*n* = 10 subjects, in triplicate).

The impact of the reactivity and the location of the carbonyl group (keto group) were investigated by comparing the two C9-length aroma compounds: nonanal and 2-nonanone. Figure 3A,B shows averaged *in vivo* aldehyde (nonanal) and ketone’s (2-nonanone) release and the dynamic sensory “green” perceived intensity curves from aqueous model systems.

The *in vivo* aroma release curves for lupin-free samples and those with nonanal and 2-nonanone (Figure 3 A) displayed distinct profiles despite sharing the same chain length. The lower polarity of the ketone’s carbonyl bond and the relocation of the keto group from the middle (2-nonanone) to the edge (nonanal) of the molecule resulted in a significant reduction of AUC_R by 73.52% and *I*_max__R by 72.25%. While no significant differences were observed in *T*_max_ values, nonanal exhibited a slower release (later *T*_max__R, see Table 2) compared to 2-nonanone. The decreased AUC_R and *I*_max__R suggested limited or reduced nonanal release over time.

Ketones, chemically less reactive than aldehydes,^51^ differ structurally by the position of their carbonyl group within the molecule, influencing their *in vivo* aroma release (Figure 3 A). Aldehydes form both reversible and irreversible bonds, while ketones predominantly bind through weaker hydrophobic interactions.^9^ Their carbonyl groups are less positively charged due to alkyl group electron donation,^52^ and their proximity may promote steric hindrance, limiting access to protein binding sites.^26,29^ This spatial configuration results in less precise fitting on the protein’s binding sites,^53,54^ indicating an increased *in vivo* release, as observed in Figure 3 A.

The present findings are consistent with prior *in vitro* investigations involving soy, whey, and myofibrillar proteins with C_5_ and C_9_ compounds.^54−56^ These studies emphasized the steric hindrance effect of ketones, indicating an increase in the free energy of association with each relocation of the carbonyl group along the chain.^56^ Furthermore, Shen et al.^55^ observed a marginally higher Stern–Volmer quenching constant for 2-pentanone compared to 3-pentanone, suggesting restricted access of 3-pentanone to hydrophobic binding sites due to the steric hindrance effect of the keto group.

With the introduction of protein to 2-nonanone samples, the AUC_R of LPI + 2-nonanone exhibited a 16.83% decrease. Similarly, the *I*_max__R of LPI + 2-nonanone decreased by 36.84%, indicating potential interactions between the protein and aroma. Sensory results showed moderate disagreement with *in vivo* release results. In protein-free samples, the displacement of the keto group from the middle to the edge of the molecule resulted in a slight increase of both AUC_S and *I*_max__S of 21.8 and 1.87%, respectively (Table 2). Upon protein addition, LPI + 2-nonanone, AUC _S decreased by 4% and *I*_max__S by 11.05%, respectively. These results suggested that the addition of the protein hindered the “green*”* perceived intensity.

### Effect of Aroma Physicochemical Properties on the *In Vivo* Aroma Release and Perception

To delve deep into the molecular aspects of the *in vivo* aroma release and sensory perception, lingering and decay rates were calculated and are shown in Table 3.

**Table 3:** Initial Intensity (*a*), Decay Rate (*b*), Lingering Duration for All Samples and Subjects, *In Vivo* Aroma Release (PTR-ToF-MS_R), and Sensory Perception (Sensory_S) (Eq 1)^a^

	LPI + 2-nonanone			LPI + nonanal			LPI + hexanal			2-nonanone			nonanal			hexanal		
	*a*	*b*		*a*	*b*		*a*	*b*		*a*	*b*		*a*	*b*		*a*	*b*	
PTR-ToF-MS_R	9.509	0.008		3.654	0.002		3.925	0.001		11.138	0.012		3.990	0.002		4.252	0.002	
Sensory_S	7.830	0.018		14.238	0.010		8.031	0.016		7.830	0.018		18.985	0.015		11.426	0.016	
lingering	75 ± 9		ab	75 ± 8		ab	71 ± 10		c	74 ± 9		ab	88 ± 6		a	60 ± 10		ab

a Data are presented as the average of the three replicates with the standard error. Letters denote significant differences (*p* < 0.05). Treatments with the same letter are not significantly different.

As seen in Table 3, a trend was generally observed between the lingering and the aroma’s physicochemical properties (*i.e*., hydrophilicity, water solubility, and volatility) (Table 1). The aroma with the greatest volatility (i.e., hexanal) was 46.94% less persistent than the most hydrophobic compound (i.e., nonanal) (Table 3). Therefore, nonanal, characterized by its lowest water solubility and volatility among the compounds (Table 1), exhibited the most prolonged lingering effect (Table 3), surpassing 2-nonanone by 15.93%.

Likewise, in protein-free samples, 2-nonanone exhibited a faster decay rate (*b*) compared to the most water-soluble (i.e., hexanal) and least volatile compound (i.e., nonanal). With the addition of protein, both *a* and *b* [eq 1] decreased *in vivo* aroma release (PTR-ToF-MS_R) for 2-nonanone and hexanal (Table 3), possibly suggesting protein–aroma interactions.

According to the obtained results (Table 3), the largest aroma lingering effect (slow decay rate) is related to the aroma’s physicochemical properties. In this context, nonanal stands out due to its hydrophobic nature and poor water solubility, as outlined in Table 1. Consequently, among the compounds investigated (nonanal, hexanal, and 2-nonanone), nonanal exhibited a longer lingering effect.

Moreover, during oral conditions, the interplay between salivary proteins and aroma can also disrupt the distribution equilibrium of aroma compounds.^10^ Previous studies aimed to determine the drivers of oral aroma persistence by examining different aroma compounds such as esters, alcohols, terpenes, and lactones.^24,26−29^ The compound’s hydrophobicity and molecular structure have been considered primary factors.^16,26,29,58,59^ Nevertheless, it should not be overlooked the ability of saliva to metabolize certain aroma compounds such as diketones and aldehydes leading to the formation of alcohols.^10,24,57^

### Effect of Mucin Protein on the *In Vitro* Aroma Release

**Figure 4:**
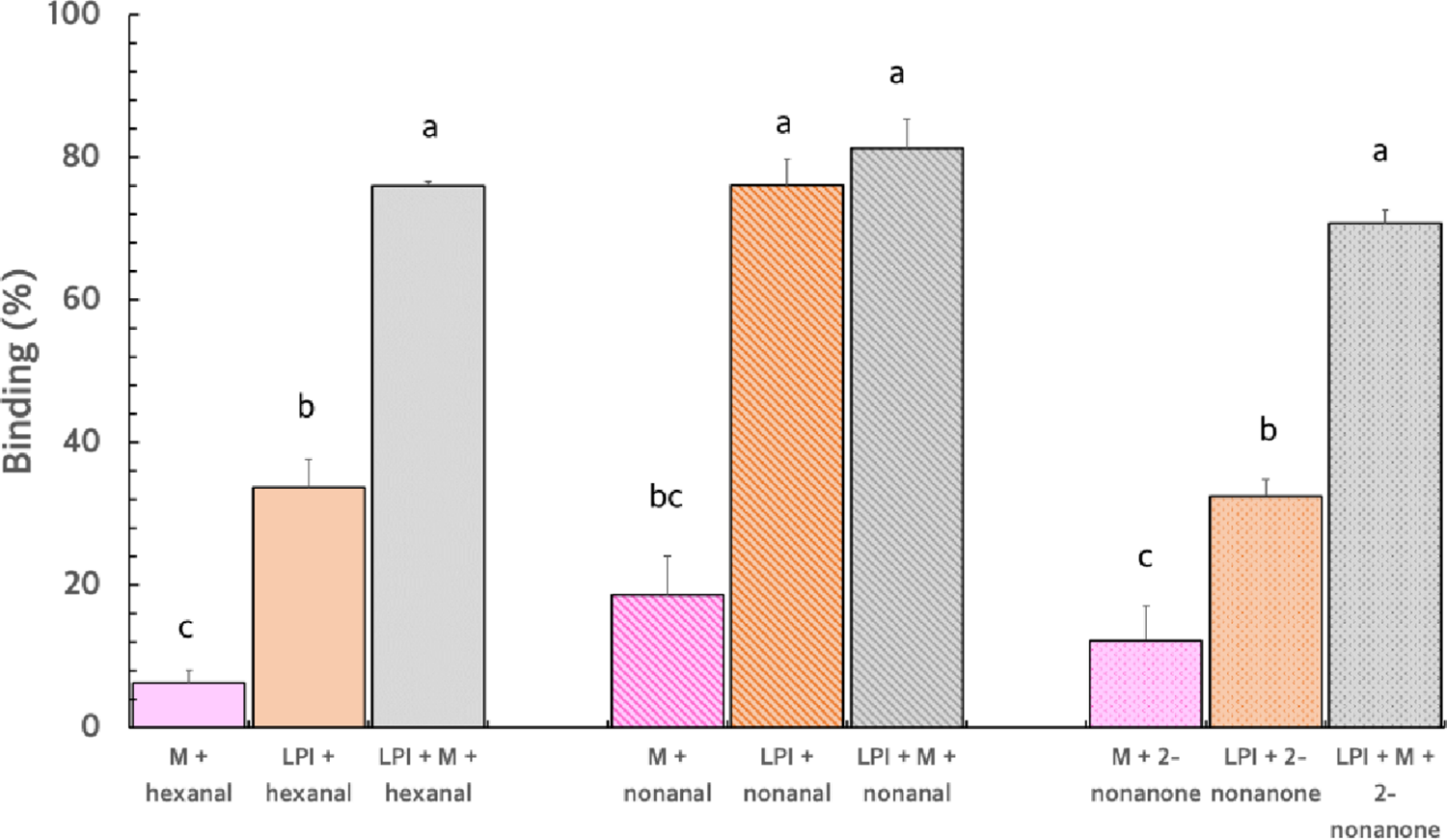
Effect of mucin on the protein-flavor binding mechanism. Binding (%) was calculated following eqs 2 and 3. Results are expressed as mean ± standard deviation. Letters denote significant differences (*p* < 0.05). Treatments with the same letter are not significantly different.

To better understand variations in the *in vivo* aroma release, it is crucial to consider potential interactions among aromas, proteins, and salivary proteins. The *in vitro* GC-MS data depicted in Figure 4 offer deeper insights into the potential interactions among aroma, proteins, and saliva. Mucin levels in the oral cavity may vary due to significant individual variability influenced by factors such as age, oral health, genetics, and other variables.^37^ Hence, this analysis utilized a minimal amount of mucin (0.01 wv%) to investigate whether even small quantities of mucin could influence the interaction between commercial LPI and aroma compounds.

As depicted in Figure 4, the GC-MS binding response (%) increased 4–12 times following the addition of mucin. Figure 4 indicates that the impact of mucin is particularly pronounced for the most volatile and hydrophilic aroma compound, which is hexanal. In contrast, the effect is less noticeable for the least volatile and most hydrophobic aroma compound, nonanal. Comparing mucin-free samples (i.e., protein and aroma) to mucin-containing samples (i.e., protein, aroma, and mucin) (Figure 4), the resulting binding effect does not simply sum up equally and proportionally. Instead, it leads to a higher binding than what would be expected solely on the basis of their individual contributions.

Mucins, rich in sialic acid residues,^60^ carry a negative charge, facilitating interactions with aldehydes through hydrogen bonding or electrostatic attractions.^10^ As observed in Figure 4, mucin exhibits a more pronounced interaction with the most hydrophobic aroma compound, nonanal. Likewise, Figure 4 suggests a synergistic effect of mucin when combined with protein and aroma. Mucins offer a finite number of binding sites (*n*),^61^ where small ligands can fit. The combined action of protein, mucin, and aroma may produce a binding with aroma that is greater than the sum of their individual effects. Although the exact mechanism of this synergistic action remains elusive, we hypothesize that the interaction of mucins with proteins may increase aroma binding by revealing the hidden hydrophobic pockets of the protein, thereby increasing the availability of the protein to interact with aroma compounds.

Limited data on aroma binding in protein-mucin mixtures exists, but synergistic behavior has been observed in protein systems.^62−64^ Ahmad et al.^62^ demonstrated cooperative effects between mucin and β-lactoglobulin, modulating the latter’s affinity and accessibility to binding sites. Similarly, Wang et al.^64^ noted synergistic effects of soy isoflavones in Whey Protein Isolate by inducing its unfolding.

The originality of this study lies in its simultaneous assessment of flavored lupin protein-based aqueous model systems, achieved by combining high-throughput *in vivo* dynamic tools with sensory profiling by using a commercial lupin protein isolate. The study underscores the influence of chain length, location of the keto group, volatility, and hydrophobicity of three aroma compounds on both *in vivo* aroma release and perception. The *in vivo* release findings indicated that longer aldehyde chains and relocation of the keto group led to a significant reduction in *I*_max__R. Upon protein addition, there was a notable decrease of *I*_max_ in both the *in vivo* aroma release and dynamic sensory perception. Due to variations in individual sensory perception and sensitivity differences between analytical techniques and human olfaction, the relationship between *in vivo* aroma release and sensory perception may not always align. The *in vivo* dynamics of aroma release and perception involve complex processes influenced by aroma physicochemical properties. Hydrophobic compounds, which are less soluble in water, showed prolonged lingering and slower decay rates. Oral processing, marked by saliva-aroma interactions, significantly affects aroma retention, although the precise mechanism remains uncertain.

Drawing conclusions about protein–aroma binding and release from exclusively three compounds and a simplified model system may not generalize to all aroma compounds or fully replicate real-world food complexity. However, studying model systems and a narrow range of compounds differing in physicochemical properties can offer valuable initial insights into the underlying mechanisms and help to identify trends and patterns in protein–aroma interactions, aiding in food design optimization.

## Supporting Information Available

Deeper understanding of the *in vivo* aroma release and perception of lupin protein-based aqueous model systems; information regarding the overview of all samples in the absence/presence of protein and absence/presence of aroma compounds (Table S1), selection of the preferred protein (*n* = 40) based on the overall taste and odor (Figure S1), attribute description of nonanal, 2-nonanone, hexanal, and lupin over the first training session (Figure S2), attribute description of lupin, nonanal, 2-nonanone, hexanal, LPI + nonanal, LPI + 2-nonanone, and LPI + hexanal over sensory sessions (Figure S3), and selected mass peaks obtained by PTR-ToF-MS (Table S2) (PDF)
