# Supplementary material for: Drivers of the In-Mouth Interaction between Lupin Protein Isolate and Selected Aroma Compounds: A Proton Transfer Reaction–Mass Spectrometry and Dynamic Time Intensity Analysis
Source: J Agric Food Chem. 2024 Apr 5;72(15):8731–41. doi: 10.1021/acs.jafc.3c08819 (PMC11036385; doi:10.1021/acs.jafc.3c08819)
Supplement: Supplementary file 1 — jf3c08819_si_001.pdf [file jf3c08819_si_001.pdf]

Drivers of the in-mouth interaction between lupin protein isolate and selected aroma compounds: a PTR-MS and Dynamic Time Intensity analysis

Cristina Barallat-Pérez[a]*, Michele Pedrotti[b], Teresa Oliviero[a], Sara Martins[a,c], Vincenzo Fogliano[a], Catrienus de Jong[d]

aDepartment of Agrotechnology and Food Science, Wageningen University & Research, 6708 WG, Wageningen, The Netherlands.

bFoundation Edmund Mach, 38098 San Michele all'Adige TN, Italy.

cAFB International EU, 5342 LZ, Oss, The Netherlands.

dWageningen Food and Biobased Research, Wageningen University & Research, 6708 WG, Wageningen, The Netherlands.

Corresponding authors:

Cristina Barallat-Pérez (refereeing, publication, and post-publication)

e-mail address: cristina1.barallatperez@wur.nl

Tel: (+31) 317482520

Catrienus de Jong (post-publication)

e-mail address: catrienus.dejong@wur.nl

Tel: (+31) 317485686

SUPPORTING INFORMATION FOR PUBLICATION

Table S1. Overview of all samples in the absence/presence of protein and absence/presence of aroma compounds.

Group	*Ingredient*	*Number of samples*	*Type of sample*	*Composition*
Only aroma compounds (absence of protein)	Hexanal/nonanal/2-nonanone	3	Control 1	5 mg/L
Only protein (absence of aroma compounds)	Lupin Protein Isolate	1	Control 2	1 wv%
Combination aroma compounds–protein	Hexanal – Lupin Protein Isolate<br>Nonanal- Lupin Protein Isolate<br>2-nonanone – Lupin Protein Isolate	3	Final sample	5 mg/L -1 wv%

Figure S1. Selection of the preferred protein (n=40) based on the overall taste and odor.

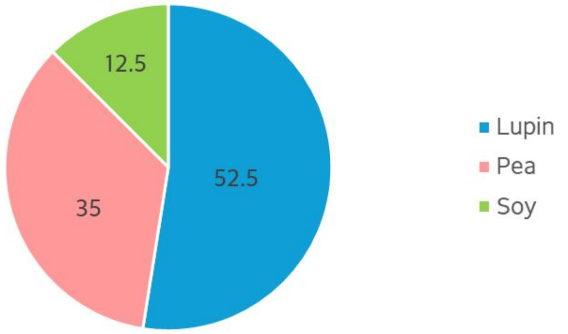

Figure S2. Attribute description of A) nonanal, B) 2-nonanone, C) hexanal, and D) lupin over the first training session.

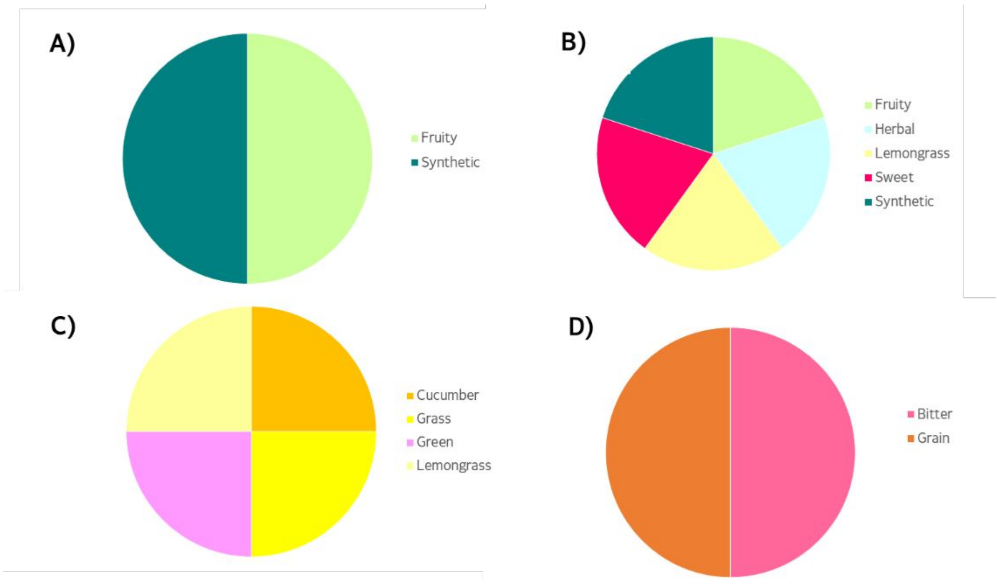

Figure S3. Attribute description of A) lupin, B) nonanal, C) 2- nonanone, D) hexanal, E) LPI +nonanal, F) LPI + 2-nonanone and G) LPI + hexanal over sensory sessions.

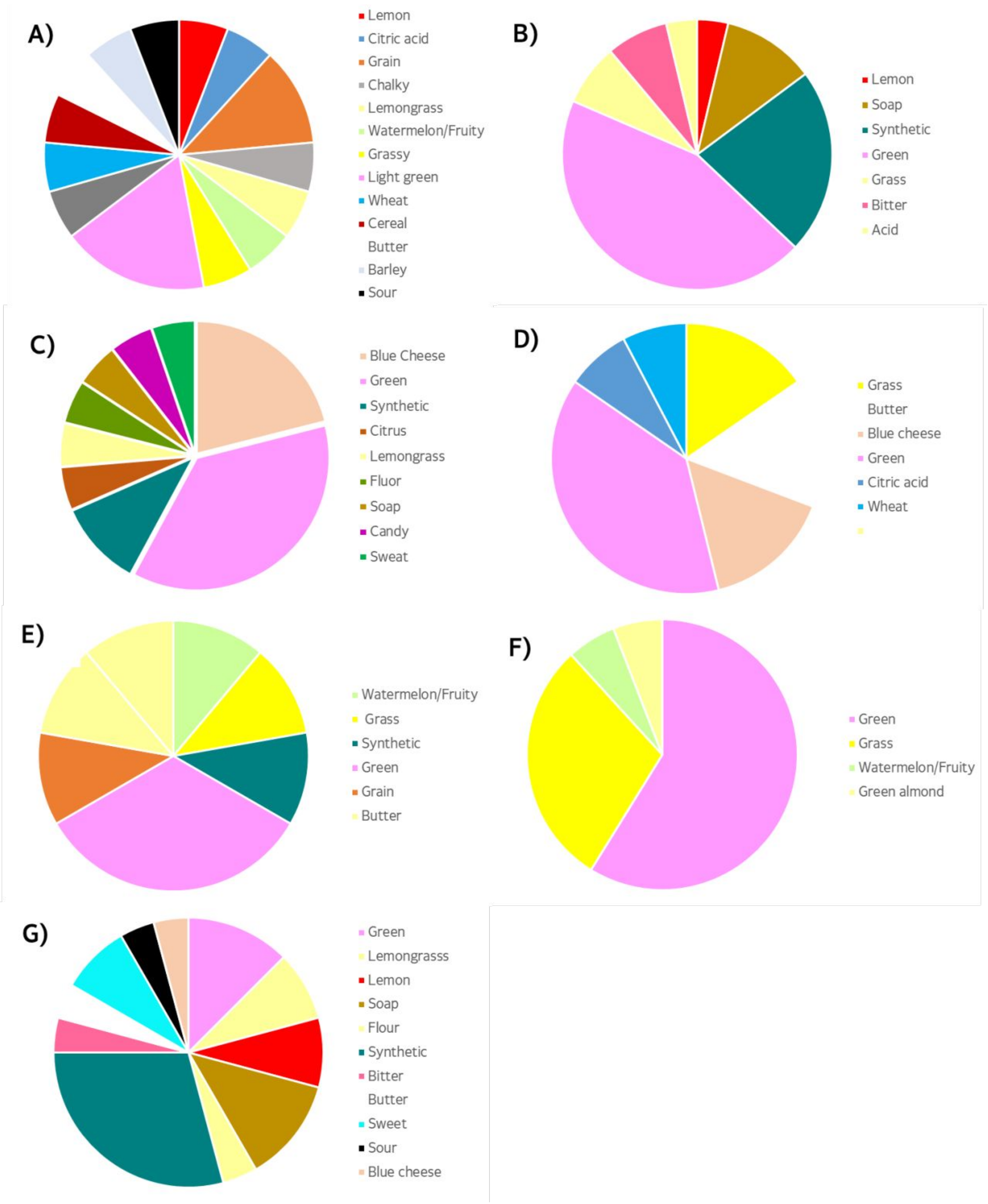

Table S2. Selected mass peaks obtained by PTR-ToF-MS [45-47].

Mass (m/z)	*Chemical formula*	*Tentative identification*
21.022	$H_3O_{18}^+$	Water molecule

45.035	$C_2H_5O^+$	Acetaldehyde
47.053	$C_2H_6OH^+$	Ethanol cluster
49.017	CH_4SH^+	Methanethiol
55.055	$C_4H_7^+$	Alkyl fragment (butanal, heptanal)
57.072	$C_4H_8H^+$	Alcohol fragment
65.061	$C_2H_5OH*H_3O+$	Ethanol cluster
73.073	$C_4H_8OH^+$	Isobutanal/2-Butanone (MEK); 2-methylpropanal
83.046	$C_5H_6OH^+$	Methyl-furan/pyran
83.093	$C_6H_{10}H^+$	Hexanal fragment
87.084	$C_5H_{10}OH^+$	2/3-Methylbutanal; C5 carbonyls; Pentenol
97.101	$C_7H1_2H^+$	C7 cycloalkanes
101.061	$C_5H_8O_2H^+$	Aromatic oxidation product; 2,3-Pentadione; Methyl-tetrahydro furanone
101.103	$C_6H_{12}OH^+$	Hexanal
115.119	$C_7H_{14}OH^+$	Heptanal; C7 carbonyls
125.096	$C_8H_{12}OH^+$	2-Nonanone/Nonanal fragment
143.158	$C_9H_{18}OH^+$	2-Nonanone/Nonanal
144.153	$C_9H_{21}NH^+$	Isotope of 2-nonanone/Nonanal
145.122	$C_8H_{16}O_2H^+$	Ethyl hexanoate/octanoic acid; 2-Pentyl propionate
